# Dermoscopic observation of Pthirus pubis

**DOI:** 10.1016/j.jdcr.2024.10.002

**Published:** 2024-10-24

**Authors:** Sam Fathizadeh, Jamison A. Harvey, David L. Swanson

**Affiliations:** a College of Medicine, University of Illinois at Chicago, Chicago, Illinois; b Department of Dermatology, Mayo Clinic, Scottsdale, Arizona

**Keywords:** infestations, lice, parasitology

## History

A 52-year-old man presented with a 1-month history of a pruritic trunk rash. Examination revealed pink papules with excoriations and wheals. Dermoscopy of the back serendipitously captured a Pthirus pubis (crab louse) recently exiting its stage III nymph exoskeleton and grasping surrounding hair. Although typically seen on the eyelids or genital hair, a genital examination was not performed, given the finding of multiple adult lice and nits visible on the back. Histology was not required for diagnosis in this case because the dermoscopic findings were definitive.

**Fig 1:**
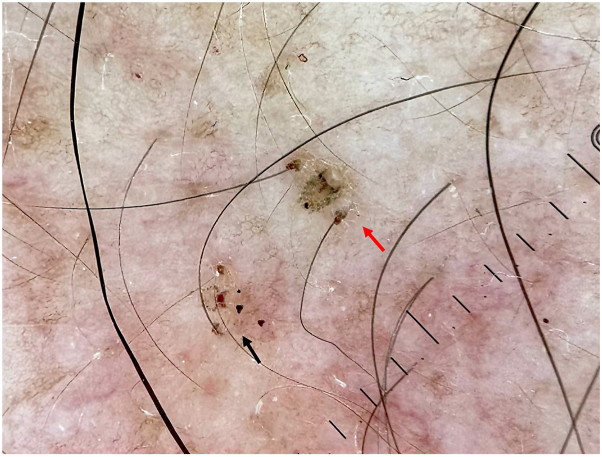
Dermoscopic image depicting the ecdysis process in a louse, where the shedding of the exoskeleton (*red arrow*) signifies a critical stage in its development cycle, transitioning toward adulthood (*black arrow*).

Pthirus pubis undergoes a life cycle beginning with eggs (nits) attached to hair shafts, followed by 3 nymphal stages before reaching adulthood.^1^^,^^2^ The dermoscopic image (Fig 1) captures ecdysis, showing the shedding of the louse's exoskeleton (red arrow) transitioning toward adulthood (black arrow). This dermoscopic finding facilitated rapid and accurate diagnosis, eliminating the need for further testing.

## Conflicts of interest

None disclosed.
